# Drug Interactions between Dolutegravir and Artemether-Lumefantrine or Artesunate-Amodiaquine

**DOI:** 10.1128/AAC.01310-18

**Published:** 2019-01-29

**Authors:** Stephen I. Walimbwa, Mohammed Lamorde, Catriona Waitt, Julian Kaboggoza, Laura Else, Pauline Byakika-Kibwika, Alieu Amara, Joshua Gini, Markus Winterberg, Justin Chiong, Joel Tarning, Saye H. Khoo

**Affiliations:** a Infectious Diseases Institute, Makerere University College of Health Sciences, Kampala, Uganda; b Department of Molecular and Clinical Pharmacology, University of Liverpool, Liverpool, United Kingdom; c Makerere University College of Health Sciences, Kampala, Uganda; d Mahidol-Oxford Tropical Medicine Research Unit, Faculty of Tropical Medicine, Mahidol University, Bangkok, Thailand

**Keywords:** dolutegravir, malaria, artemether, artesunate, lumefantrine, amodiaquine, drug-drug interactions

## Abstract

Across sub-Saharan Africa, patients with HIV on antiretrovirals often get malaria and need cotreatment with artemisinin-containing therapies. We undertook two pharmacokinetic studies in healthy volunteers, using standard adult doses of artemether-lumefantrine or artesunate-amodiaquine given with 50 mg once daily dolutegravir (DTG) to investigate the drug-drug interaction between artemether-lumefantrine or artesunate-amodiaquine and dolutegravir. The dolutegravir/artemether-lumefantrine interaction was evaluated in a two-way crossover study and measured artemether, dihydroartemisinin, lumefantrine, and desbutyl-lumefantrine over 264 h. The dolutegravir/artesunate-amodiaquine interaction was investigated using a parallel study design due to long half-life of the amodiaquine metabolite, desethylamodiaquine and measured artesunate, amodiaquine, and desethylamodiaquine over 624 h. Noncompartmental analysis was performed, and geometric mean ratios and 90% confidence intervals were generated for evaluation of both interactions. Dolutegravir did not significantly change the maximum concentration in plasma, the time to maximum concentration, and the area under the concentration-time curve (AUC) for artemether, dihydroartemisinin, lumefantrine, and desbutyl-lumefantrine, nor did it significantly alter the AUC for artesunate, dihydroartemisinin, amodiaquine, and desethylamodiaquine. Coadministration of dolutegravir with artemether-lumefantrine resulted in a 37% decrease in DTG trough concentrations. Coadministration of dolutegravir with artesunate-amodiaquine resulted in 42 and 24% approximate decreases in the DTG trough concentrations and the AUC, respectively. The significant decreases in DTG trough concentrations with artemether-lumefantrine and artesunate-amodiaquine and dolutegravir exposure with artesunate-amodiaquine are unlikely to be of clinical significance since the DTG trough concentrations were above dolutegravir target concentrations of 300 ng/ml. Study drugs were well tolerated with no serious adverse events. Standard doses of artemether-lumefantrine and artesunate-amodiaquine should be used in patients receiving dolutegravir. (This study has been registered at ClinicalTrials.gov under identifier NCT02242799.)

## Abstract (precis)

Across sub-Saharan Africa, patients with HIV on antiretrovirals often get malaria and need cotreatment with artemisinin-containing therapies. We undertook two pharmacokinetic studies in healthy volunteers, using standard adult doses of artemether-lumefantrine or artesunate-amodiaquine given with 50 mg once daily dolutegravir (DTG) to investigate the drug-drug interaction between artemether-lumefantrine or artesunate-amodiaquine and dolutegravir.

## INTRODUCTION

Over 90% of malaria cases occur in sub-Saharan Africa (SSA), the region with the greatest burden of HIV (1). Drug-drug interactions (DDIs) between antiretrovirals and artemisinin-based combination therapies (ACTs) frequently occur and may affect the clinical effectiveness of commonly utilized antimalarials artemether-lumefantrine (AL) and artesunate-amodiaquine (AS-AQ) (2–4). The likely adoption of dolutegravir (DTG) in preferred first-line antiretroviral therapy (ART) regimens (4) makes a DDI study with antimalarials an urgent priority. Dolutegravir is predominantly metabolized via UDP glucuronyl transferase 1A1 (UGT 1A1), with minor input from cytochrome P450 3A4 (CYP3A4), which suggests minimal DDI potential as a perpetrator drug (5).

Both artemether (ARM) and lumefantrine (LF) are predominantly metabolized via CYP3A4, CYP2B6, CYP2C9, and CYP2C19 to active metabolites dihydroartemisinin (DHA) and desbutyl-lumefantrine (DBL), respectively. Artesunate (AS) is a prodrug and substrate of CYP2A6 and undergoes rapid hydrolysis to DHA, while amodiaquine (AQ) is extensively metabolized by CYP 2C8 to its active metabolite, *N*-desethylamodiaquine (DEAQ) (8). Coadministration of artemether-lumefantrine with inducers of CYP3A4 results in significant reductions in artemether and dihydroartemisinin exposures (6, 8). Similarly, clinically significant DDIs with ritonavir-boosted protease inhibitor ART regimens have been reported (7). However, data for dolutegravir interactions with antimalarial therapies are lacking. We investigated the pharmacokinetic (PK) interactions between dolutegravir with artemether-lumefantrine or artesunate-amodiaquine and assessed the safety and tolerability of the drug combinations.

(This research was presented in part at the Conference on Retroviruses and Opportunistic Infections, 4 to 7 March 2018, Boston, MA [abstr. 459].)

## RESULTS

Forty-eight participants were enrolled into both studies: 18 in study A and 30 in study B. In study A, two participants withdrew consent, and another two were terminated from the study due to nonadherence to study procedures. In study B, one participant was withdrawn due to safety concerns, three participants withdrew consent, and one was terminated from the study due to nonadherence to study procedures. Statistical analysis was performed on 39 subjects who completed study procedures and their demographic variables are presented in Table 1 .

**TABLE 1:** Participant median baseline demographic variables^*a*^

Parameter (*n* = 39)	Median (IQR)			
	Study A (*n* = 14)		Study B (*n* = 25)	
	Sequence 1 (*n* = 7)	Sequence 2 (*n* = 7)	Arm 1 (*n* = 13)	Arm 2 (*n* = 12)
Age (yrs)	29 (21–32)	25 (23–29)	24 (23–28)	30.5 (23.5–34)
Wt (kg)	55.5 (54–64)	59 (54–62)	59.5 (57–65)	60.25 (58–68.25)
BMI (kg/m^2^)	21.1 (17–22.8)	21.2 (20.1–21.5)	21.4 (19.8–24.5)	20.5 (18.95–24.5)
Hemoglobin (g/dl)	15.1 (13.3–17.7)	14.7 (13.7–17.0)	15.2 (13.5–16.2)	15.3 (14.5–16.3)
ALT (IU/liter)	12 (9–20)	15 (12–17)	15 (13–19)	18 (13–20)
Total bilirubin (mg/dl)	0.7 (0.4–0.9)	0.6 (0.4–1.1)	0.5 (0.3–0.7)	0.6 (0.35–1.65)
Urea (mg/dl)	7 (6–9)	7 (6–9)	7 (5–8)	8 (6.5–11)
Creatinine (mg/dl)	0.76 (0.59–0.93)	0.75 (0.62–0.79)	0.82 (0.67–0.92)	0.85 (0.69–0.92)
Corrected QT interval (ms)	387 (378–407)	415 (397–429)	396 (369–408)	400.5 (373.5–414.5)

a Note that QT is used in the Fridericia formula. Interquartile ranges (IQR) are indicated in parentheses. BMI, body mass index; ALT, alanine transaminase.

### Antimalarial pharmacokinetics. (i) Effect of dolutegravir on artemether-lumefantrine pharmacokinetics (study A).

In study A, 14 participants received artemether-lumefantrine (7, sequence 1; 7, sequence 2). The PK profiles for artemether/dihydroartemisinin (0 to 24 h), lumefantrine/desbutyl-lumefantrine (0 to 264 h), and associated PK parameters are presented in Table 2 .

**TABLE 2:** Artemether-lumefantrine and artesunate-amodiaquine pharmacokinetic parameters alone and in combination with dolutegravir^*a*^

Study and parameter	GM (90% CI)		GMR (90% CI)
	Alone	In combination^*b*^	
Study A (*n* = 14)			
ARM			
* C*_max_ (ng/ml)	31.93 (20.60–43.26)	27.88 (10.30–45.47)	0.87 (0.67–1.14)
* T*_max_ (h)	2.03 (1.64–2.43)	2.16 (1.75–2.56)	1.06 (0.84–1.34)
* *AUC_0–_*_t_* (ng ⋅ h/ml)	129.6 (79.35–179.8)	136.4 (60.29–212.6)	1.05 (0.84–1.32)
* *CL/F (liters/h)	617.4 (307.4–927.3)	586.3 (83.20–1089)	0.95 (0.76–1.19)
* t*_1/2_ (h)	4.92 (3.27–6.57)	7.28 (5.09–9.48)	**1.57 (1.09–2.27)**
* *DHA			
* C*_max_ (ng/ml)	110.4 (92.86–128.0)	89.91 (71.07–108.7)	0.81 (0.64–1.03)
* T*_max_ (h)	2.31 (1.98–2.64)	2.70 (1.99–3.42)	1.17 (0.92–1.49)
* *AUC_0–_*_t_* (ng ⋅ h/ml)	389.3 (344.5–434.0)	357.3 (274.9–439.6)	0.92 (0.79–1.07)
* t*_1/2_ (h)	2.54 (2.05–3.03)	3.01 (1.33–4.69)	1.38 (0.99–1.93)
* *LF			
* C*_max_ (ng/ml)	9,976 (8,318–11,633)	11203 (9533–12873)	1.12 (0.97–1.29)
* T*_max_ (h)	3.92 (2.49–5.35)^*c*^	6.48 (1.43–11.54)^*d*^	**1.65 (1.02–2.69)**
* *AUC_0–_*_t_* (ng ⋅ h/ml)	389,350 (333,608–445,092)	429736 (379,911–479,561)	1.10 (0.96–1.27)
* *CL/F (liters/h)	1.23 (1.06–1.41)	1.12 (0.94–1.29)	0.91 (0.79–1.04)
* t*_1/2_ (h)	83.44 (76.37–90.51)	86.13 (76.46–95.81)	1.03 (0.90–1.18)
* *DBL			
* C*_max_ (ng/ml)	51.75 (37.50–66.00)	49.95 (41.54–58.35)	0.97 (0.79–1.18)
* T*_max_ (h)	4.78 (3.43–6.12)	9.52 (4.48–14.56)^*c*^	**3.00 (2.06–4.36)**
* *AUC_0–_*_t_* (ng ⋅ h/ml)	6299 (4,804–7,796)	6049 (5,235–6,862)	0.96 (0.80–1.15)
* t*_1/2_ (h)	141.6 (130.3–152.9)	162.1 (134.6–189.6)	1.15 (1.00–1.31)
			
Study B (*n* = 25)			
* *ARS			
* C*_max_ (ng/ml)	61.29 (41.54–81.04)	52.01 (31.70–72.33)	0.85 (0.56–1.28)
* T*_max_ (h)	1.17 (0.78–1.56)	1.66 (1.14–2.17)	1.41 (1.01–1.98)
* *AUC_0–_*_t_* (ng ⋅ h/ml)	128.4 (90.81–165.9)	115.7 (83.22–148.2)	0.90 (0.59–1.37)
* *CL/F (liters/kg/h)	31.16 (22.12–40.19)	34.57 (18.40–50.74)	1.10 (0.72–1.70)
* t*_1/2_ (h)	1.85 (0.50–3.20)	1.17 (0.74–1.60)	0.63 (0.32–1.23)
* *DHA			
* C*_max_ (ng/ml)	217.7 (157.4–278.0)	290.4 (197.3–383.6)	1.33 (0.88–2.02)
* T*_max_ (h)	1.58 (1.16–2.00)	2.02 (1.52–2.52)	1.28 (0.91–1.79)
* *AUC_0–_*_t_* (ng ⋅ h/ml)	788.3 (622.1–954.4)	946.8 (760.2–1133)	1.20 (0.89–1.62)
* t*_1/2_ (h)	2.22 (0.89–5.32)	1.60 (1.38–1.81)	0.72 (0.46–1.15)
* *AQ			
* C*_max_ (ng/ml)	17.79 (14.91–20.68)	19.17 (15.95–22.39)	1.08 (0.84–1.38)
* T*_max_ (h)	2.36 (1.06–3.65)	1.97 (1.43–2.51)	0.84 (0.55–1.27)
* *AUC_0–_*_t_* (ng ⋅ h/ml)	256.1 (222.5–289.8)	225.0 (198.9–251.1)	0.88 (0.72–1.07)
* *CL/F (liters/kg/h)	39.04 (31.11–46.97)	44.44 (38.75–50.13)	1.12 (0.93–1.38)
* t*_1/2_ (h)	15.83 (14.29–17.37)	14.79 (12.62–16.97)	0.93 (0.78–1.12)
* *DEAQ			
* C*_max_ (ng/ml)	394.0 (325.9–462.0)	385.6 (346.8–424.3)	0.98 (0.79–1.21)
* T*_max_ (h)	2.68 (1.88–3.49)	3.38 (2.41–4.36)	1.26 (0.89–1.78)
* *AUC_0–_*_t_* (ng ⋅ h/ml)	31,493 (28721–34265)	26,943 (22913–30973)	0.86 (0.70–1.05)
* t*_1/2_ (h)	243.7 (230.5–256.9)	182.5 (141.5–223.5)	0.75 (0.53–1.06)
			
DTG			
* *Study A (*n* = 14)			
* C*_24_ (ng/ml)	2,456 (2,062–2,851)	1,543 (1,122–1,965)	**0.63 (0.48–0.82)**
* C*_max_ (ng/ml)	5,018 (4,512–5,525)	5,216 (46234–5809)	1.04 (0.92–1.18)
* T*_max_ (h)	3.94 (1.41–6.46)	3.00 (1.89–4.10)	0.90 (0.66–1.24)
* *AUC_0–24_ (ng ⋅ h/ml)	78,753 (70,615–86,891)	73,738 (63,420–84,057)	0.94 (0.86–1.02)
* *CL/F (liters/h)	0.63 (0.53–0.74)	0.68 (0.59–0.76)	1.07 (0.99–1.16)
* t*_1/2_ (h)	24.29 (15.73–32.84)	13.01 (10.00–16.03)	**0.49 (0.31–0.76)**
* *Study B (*n* = 12)			
* C*_24_ (ng/ml)	2,174 (1,567–2,781)	1,272 (1,025–1,518)	**0.58 (0.50–0.69)**
* C*_max_ (ng/ml)	5,114 (4,562–5,667)	4667 (3940–5393)	0.91 (0.80–1.04)
* T*_max_ (h)	3.7 (2.7–4.7)	2.7 (1.8–3.5)	0.72 (0.50–1.04)
* *AUC_0–24_ (ng ⋅ h/ml)	77,936 (67,805–88,068)	59,491 (52,480–66,502)	**0.76 (0.69–0.84)**
* *CL/F (liters/h)	0.64 (0.54–0.74)	0.84 (0.73–0.95)	1.31 (1.18–1.45)
* t*_1/2_ (h)	16.21 (12.72–19.70)	13.31 (11.30–15.32)	**0.76 (0.61–0.95)**

a ARM, Artemether; DHA, dihydroartemisinin; LF, lumefantrine; DBL, desbutyl-lumefantrine; ARS, artesunate; AQ, amodiaquine; DEAQ, desethylamodiaquine. For study A, the time of the last measurable concentration (*t*) = 11.8 h (7.1 to 16.5), ARM alone; 16.5 h (8.5 to 24.5), ARM+DTG; 11.7 h (11.2 to 12.1), DHA alone; 13.3 h (9.0 to 17.4), DHA+DTG; and 264 h LF alone, LF+DTG, DBL alone, and DBL+DTG. For study B, the time of the last measurable concentration (*t*) = 9.15 h (7.95 to 10.34), AS alone; 7.44 h (6.22 to 8.67), AS+DTG; 11.63 h (11.12 to 12.14), DHA alone; 11.60 h (11.05 to 12.15), DHA+DTG; 69.16 h (63.74 to 74.58), AQ alone; 60.81 (54.92 to 66.69), AQ+DTG; and 624 h (DEAQ alone) and 509.96 h (430.24 to 589.68), DEAQ+DTG. Boldfacing indicates significant interactions observed in the study.

b AL or AS-AQ plus DTG.

c One subject had a *T*_max_ at 0 h.

d One subject had a *T*_max_ at 48 h as a secondary peak.

When given with dolutegravir, artemether’s *C*_max_ decreased by 13% (geometric mean ratio [GMR], 0.87; 90% confidence interval [CI], 0.67 to 1.14) after approximately 2 h, with a 5% increase in the area under the concentration-time curve from time 0 to the last measurable concentration (AUC_0–_*_t_* ; GMR, 1.05; 90% CI, 0.84 to 1.32). The active metabolite dihydroartemisinin peak concentrations decreased by 19% (GMR, 0.81; 90% CI, 0.64 to 1.03) after 2.3 h, with a decrease of 8% in AUC_0–_*_t_* (GMR, 0.92; 90% CI, 0.79 to 1.07). Artemether and dihydroartemisinin were eliminated from plasma with average half-lives of 5 and 2.5 h, respectively.

Similarly lumefantrine showed peak concentrations approximately 4 h after drug administration, with a 12% increase in *C*_max_ (GMR, 1.12; 90% CI, 0.97 to 1.29) and 10% increase in AUC_0–_*_t_* (GMR, 1.10; 90% CI, 0.96 to 1.27). The lumefantrine metabolite desbutyl-lumefantrine had a 3% decrease in *C*_max_ (GMR, 0.97; 90% CI, 0.79 to 1.18) and a 4% decrease in AUC_0–_*_t_* (GMR, 0.96; 90% CI, 0.80 to 1.15), representing approximately 1.7% of the total circulating lumefantrine. Both lumefantrine and desbutyl-lumefantrine had prolonged mean elimination half-lives of approximately 83 and 142 h, respectively.

The GMR for each antimalarial and metabolite are presented in Table 2. Coadministration of artemether-lumefantrine with dolutegravir did not significantly alter the *C*_max_, the AUC_0–_*_t_*, or the clearance of artemether, lumefantrine, or their metabolites. Furthermore, the time of the last measurable concentration (*t*) for all artemether-lumefantrine components did not significantly differ when administered alone or in combination with DTG. Interestingly, the terminal elimination half-life of artemether was increased in combination with DTG (GMR, 1.57; 90% CI, 1.09 to 2.27), but this appeared to be largely driven by two subjects who had detectable artemether levels beyond 24 h in combined phase. The analysis of variance (ANOVA) showed no evidence of a significant sequence or period effect upon artemether-lumefantrine PK.

### (ii) Effect of dolutegravir on artesunate-amodiaquine pharmacokinetics (study B).

In study B, 25 participants received artesunate-amodiaquine. Thirteen subjects in arm 1 were administered artesunate-amodiaquine alone, and 12 subjects in arm 2 were administered artesunate-amodiaquine with dolutegravir. The PK profiles for artesunate/dihydroartemisinin (0 to 12 h), amodiaquine/*N*-desethylamodiaquine (0 to 624 h), and associated PK parameters are presented in Table 2.

When given with dolutegravir, artesunate maximum concentrations reduced by 15% (GMR, 0.85; 90% CI, 0.56 to 1.28) within 1.2 h, with an overall decrease in AUC_0–_*_t_* of 10% (GMR, 0.90; 90% CI, 0.59 to 1.37) compared to artesunate-amodiaquine alone. Dihydroartemisinin exposures were, on average, 6-fold higher than the corresponding artesunate AUC_0–_*_t_* values. Artesunate and dihydroartemisinin had geometric mean half-lives of 1.9 and 2.2 h, respectively. Similarly, amodiaquine was rapidly absorbed (time to reach maximum concentration [*T*_max_] = 2.4 h) and was detectable in plasma for approximately 70 h postdose; the overall amodiaquine AUC_0–_*_t_* was 256.1 ng ⋅ h/ml (222.5 to 289.8). Amodiaquine was rapidly and extensively converted to *N*-desethylamodiaquine (*T*_max_ = 2.7 h); *N*-desethylamodiaquine exposures were ∼122-fold higher than amodiaquine and persisted in plasma for up to 624 h postdose (terminal elimination half-life, ca. 9 to 18 days). Coadministration of dolutegravir with artesunate-amodiaquine did not significantly alter the AUC_0–_*_t_* for artesunate (GMR, 0.90; 90% CI, 0.59 to 1.37), dihydroartemisinin (GMR, 1.20; 90% CI, 0.89 to 1.62), amodiaquine (GMR, 0.88; 90% CI, 0.72 to 1.07), or *N*-desethylamodiaquine (GMR, 0.86; 90% CI, 0.70 to 1.05).

### (iii) Dolutegravir pharmacokinetics.

**FIG 1:**
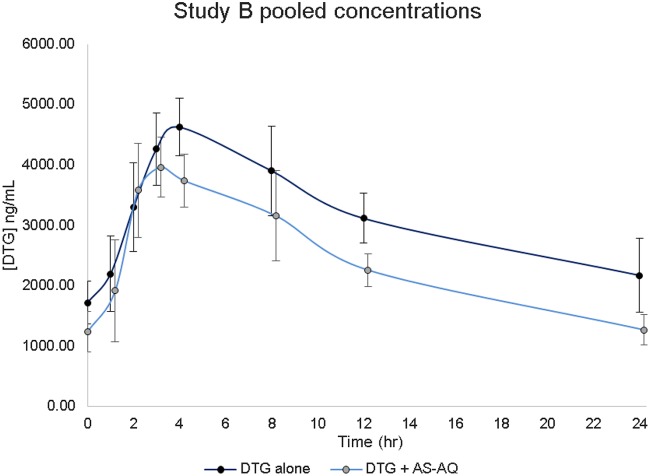
Study A (dolutegravir ± artemether-lumefantrine) concentration-time profiles (means plus standard deviations).

**FIG 2:**
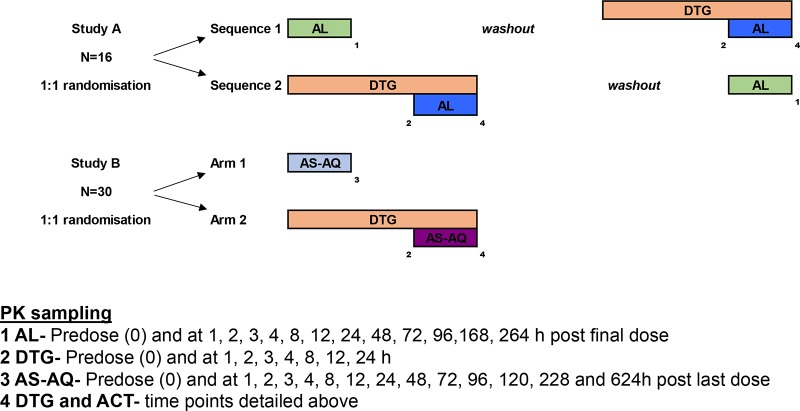
Study B (dolutegravir ± artesunate-amodiaquine) concentration-time profiles (means plus standard deviations).

Dolutegravir was administered with and without antimalarials to 14 participants in study A (7, sequence 1; 7, sequence 2) and 12 participants in study B (arm 2). Pooled dolutegravir and antimalarial concentration-time profiles (means plus standard deviations) from 0 to 24 h for studies A and B are depicted in Fig. 1 and 2, respectively.

### (iv) Effect of artemether-lumefantrine on dolutegravir pharmacokinetics (study A).

In study A, dolutegravir *C*_max_ increased by 4% (GMR, 1.04; 90% CI, 0.92 to 1.18) approximately 3.9 h postdose, with an overall AUC_0–24_ decrease of 6% (GMR, 0.94; 90% CI, 0.86 to 1.02) compared to steady-state dolutegravir alone. Coadministration of dolutegravir with artemether-lumefantrine resulted in a 37% decrease in the dolutegravir concentration at 24 h (*C*_24_ ; GMR, 0.63; 90% CI, 0.48 to 0.82). No significant changes were observed in dolutegravir AUC_0–24_ or *C*_max_ when dolutegravir was administered with artemether-lumefantrine (Table 2). The ANOVA revealed no significant sequence effect upon dolutegravir PK. Furthermore, there was no significant period effect (DTG alone versus DTG+AL) for the dolutegravir *C*_24_ in both arms.

### (v) Effect of artesunate-amodiaquine on dolutegravir pharmacokinetics (study B).

In study B, dolutegravir *C*_max_ decreased by 9% (GMR, 0.91; 90% CI, 0.80 to 1.04) approximately 3.7 h postdose, with an overall AUC_0–24_ decrease of 24% (GMR, 0.76; 90% CI, 0.69 to 0.84). Coadministration of dolutegravir with artesunate-amodiaquine resulted in a significant decrease of approximately 42 and 24% in the dolutegravir *C*_24_ and AUC_0–24_, respectively, as presented in Table 2.

### Safety.

No deaths or serious adverse events were reported during the study. In all, 111 adverse events were observed and reported, with 80 adverse events judged as having a temporal relationship with study medication. All adverse events were grade 1 or 2 in severity. Gastrointestinal adverse events were more common among participants receiving artesunate-amodiaquine. No clinically significant changes were observed during safety assessments (laboratory blood assessments, ECG, or vital signs).

## DISCUSSION

This is the first study to examine for drug interactions between dolutegravir with artemether-lumefantrine and artesunate-amodiaquine. We found no significant impact of dolutegravir upon drug exposure of either antimalarial regimen, suggesting that standard doses of artemether-lumefantrine and artesunate-amodiaquine should be used when coadministered with dolutegravir. These findings are important given the increasing use of dolutegravir in first-line antiretroviral therapy regimens. The results confirm the low propensity for dolutegravir to perpetrate clinically significant DDIs, as judged by *in vitro* observations of minimal effects on drug transporters and cytochrome P450 enzymes (5, 13).

We observed that artemether-lumefantrine was not associated with any significant change in dolutegravir exposure parameters (*C*_max_ and AUC_0–24_). However, dolutegravir *C*_24_ was 37% lower with artemether-lumefantrine than when given alone. The reasons for this are unclear but may have been driven in part by an unexplained rise in dolutegravir *C*_24_ in some participants. Additional intake of dolutegravir prior to the last sampling point at 24 h was unlikely, since subjects were instructed not to take the next dose before this time point and were issued with an exact number of pills, which precluded such additional intake.

With artesunate-amodiaquine, we observed an unexplained statistically significant reduction of 42 and 24% for dolutegravir *C*_24_ and AUC_0–24_, respectively. However, in all subjects who received DTG with artemether-lumefantrine or artesunate-amodiaquine, the dolutegravir *C*_trough_ was comparable to or above 1,100 ng/ml (10), the mean *C*_trough_ observed in prior dolutegravir phase 3 adult trials. The target minimum effective concentrations for dolutegravir are unknown, although a DTG protein-adjusted effective concentration (EC_90_) above 300 ng/ml has been proposed (11, 12). In a phase II study, *C*_trough_ concentrations over 324 ng/ml after 10 days of DTG monotherapy were associated with virological efficacy (13). All subjects in our study had *C*_trough_ concentrations exceeding these targets, suggesting that the modest pharmacokinetic changes observed have unlikely clinical significance, especially given the short duration of antimalarial therapy.

Notably, dolutegravir concentrations in this study of black African healthy volunteers were somewhat higher than previously reported in Caucasians where exposures of 4,560 ng/ml and 7,080 ng ⋅ h/ml for *C*_max_ and AUC_0–_*_t_*, respectively, were observed (11). However, it should be noted that in our study, dolutegravir was dosed with a moderate fat meal, and concentrations observed are consistent with reports on the food effect upon DTG bioavailability (14).

We observed a 65% increase in *T*_max_ for lumefantrine (GMR, 1.65; 90% CI, 1.02 to 2.69) and a 3-fold increase in *T*_max_ for desbutyl-lumefantrine (GMR, 3.00; 90% CI, 2.06 to 4.36) during dolutegravir/artemether-lumefantrine coadministration. The reasons for the increase in *T*_max_ for lumefantrine and desbutyl-lumefantrine are unclear, but the observed differences are very unlikely to be clinically significant.

This intensive pharmacokinetic study evaluated the drug-drug interactions using a two-way crossover study design with a 21-day washout period and a parallel study design for dolutegravir/artemether-lumefantrine and dolutegravir/artesunate-amodiaquine, respectively. While a crossover study design would be theoretically ideal for investigating both artemisinin containing therapies in combination with dolutegravir, the amodiaquine active metabolite, *N*-desethylamodiaquine, has an extensive terminal half-life of approximately 9 to 18 days; therefore, it was not feasible to undertake a crossover design for this arm of the study since the washout period between the two phases would exceed two months, risking subject attrition. Furthermore, the crossover study design was chosen to allow for a two-way analysis for potential drug interactions between dolutegravir and artemether-lumefantrine. However, the elimination half-life for lumefantrine active metabolite desbutyl-lumefantrine was unexpectedly increased to ∼142 h, suggesting that the washout period for desbutyl-lumefantrine may not have been sufficiently long. Although this finding highlights a limitation in our study, the analysis of variance found no period or sequence effects upon artemether-lumefantrine pharmacokinetics.

Dolutegravir is a weak inhibitor of organic cation transporter 2 (OCT2) and multidrug and toxin extrusion 1 (MATE1) transporter with reported interactions with substrates of both transporters. However, neither lumefantrine nor desbutyl-lumefantrine is a substrate of OCT2 and MATE1. It remains unclear by which mechanism dolutegravir might have influenced the observed lumefantrine/desbutyl-lumefantrine pharmacokinetics.

The combination of dolutegravir with artemether-lumefantrine and artesunate-amodiaquine was well tolerated. Nausea, the most common study drug-related adverse event, was reported predominantly in the artesunate-amodiaquine arm. This safety profile was consistent with the approved drug labeling (14–16).

In conclusion, standard treatment regimens of artesunate-amodiaquine and of artemether-lumefantrine should be prescribed when treating malaria in HIV-infected patients receiving a dolutegravir-based antiretroviral regimen.

## MATERIALS AND METHODS

### Ethics.

The study was approved by the Joint Clinical Research Centre Institutional Review Board, Kampala, Uganda, and the University of Liverpool Research Ethics Committee, Liverpool, United Kingdom, and registered at ClinicalTrials.gov (NCT02242799). The study was conducted in compliance with International Council for Harmonization Good Clinical Practice guidelines, the current ethical principles in the Declaration of Helsinki, and applicable local regulatory requirements.

### Study design.

**FIG 3:**
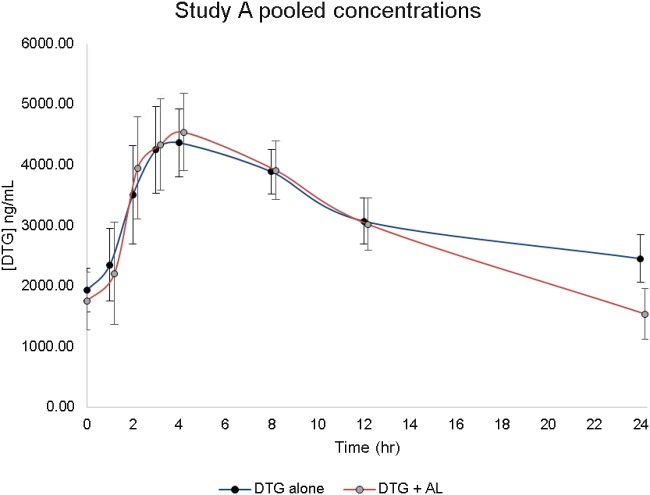
Study design. Two intensive PK studies using standard treatment doses of artemether-lumefantrine and artesunate-amodiaquine with 50 mg of dolutegravir given once daily.

Two open-label, fixed sequence studies between dolutegravir (Tivicay; ViiV Healthcare, Research Triangle Park, NC) given at 50 mg once daily and artemether-lumefantrine (Coartem; Novartis Pharma AG, Basel, Switzerland) (study A) or artesunate-amodiaquine (Winthrop; Sanofi-Aventis, Casablanca, Morocco) (study B) were conducted at the Infectious Diseases Institute, Kampala, Uganda (Fig. 3). Inclusion of 16 subjects in study A was calculated to have a >80% power to detect a change in AUC outside U.S. Food and Drug Administration (FDA) limits for bioequivalence (with the 90% CI for AUC falling within 80 to 125%) for dolutegravir and lumefantrine (assuming a coefficient of variation of ≤30%) and to detect a ≥32% change in dihydroartemisinin levels. Including 30 subjects in study B would yield an 80% power to detect an AUC difference of >25% to 30% (DTG and DEAQ), and a ≥42% change for dihydroartemisinin.

### Eligibility criteria.

Consenting healthy adults (≥18 years) weighing >40 kg, without malaria and HIV, were eligible to participate if they were willing to use mosquito bed nets and able to comply with study procedures. Subjects were excluded if they had evidence of significant systemic disease, serum liver transaminases greater than three times the upper limits of normal, serum creatinine grater than two times the upper limits of normal, positive hepatitis B surface antigen, and evidence of QT prolongation on ECG exceeding 450 ms (men) or 470 ms (women) and were taking medications, which are well-known inhibitors or inducers of CYPs or glucuronyltransferase UGT1A1. Pregnant or breastfeeding women and female volunteers unwilling to use reliable contraception during the study were also excluded.

### Dosing and sampling.

**(i) Study A (artemether-lumefantrine).** Study A used a random sequence two-way crossover study design with participants randomized to sequence 1 or sequence 2. In sequence 1, participants received six doses of oral artemether-lumefantrine tablets (80/480 mg twice daily taken with food) for 3 days (regimen used for treatment of uncomplicated malaria) with PK sampling predose (0 h) and 1, 2, 3, 4, 8, 12, 24, 48, 72, 96, 168, and 264 h after the final dose. After a 21-day washout, selected from the expected terminal half-lives of lumefantrine and desbutyl-lumefantrine, they received dolutegravir 50 mg once daily alone for 6 days with PK sampling on day 6 predose (0 h) and 1, 2, 3, 4, 8, 12, and 24 h postdosing. Subsequently, they received 3 days of twice daily AL plus dolutegravir, with PK sampling predose (0 h) and 1, 2, 3, 4, 8, 12, 24, 48, 72, 96, 168, and 264 h after the final doses of both drugs using the sampling time points described. In sequence 2, participants received the dolutegravir and DTG-AL combination with PK sampling as detailed for sequence 1, followed by artemether-lumefantrine alone after the 21-day washout period.

**(ii) Study B (artesunate-amodiaquine).** Study B used a parallel group design due to the long terminal half-life of approximately 9 to 18 days for the amodiaquine active metabolite *N*-desethylamodiaquine. Participants were randomized to receive AS-AQ (4 mg/kg AS, 10 mg/kg AQ) once daily for 3 days with PK sampling predose (0 h) and 1, 2, 3, 4, 8, 12, 24, 48, 72, 96, 120, 228, and 624 h after the last dose (arm 1) or dolutegravir for 7 days with PK sampling at predose (0 h) and at 1, 2, 3, 4, 8, 12, and 24 h after the last dose, followed by AS-AQ once daily, together with dolutegravir 50 mg once daily for 3 days, with PK sampling after the last dose of both drugs using the sampling time points listed (arm 2).

All study drugs prior to intensive PK sampling were administered after an overnight fast. A standard moderate fat breakfast was provided after the predose (0 h) sampling.

### Safety assessments.

At screening, a medical history, physical examination, urine pregnancy test, rapid malaria and HIV tests, and safety bloods (hemoglobin, white cell count, platelets, urea, creatinine, electrolytes, and ALT [alanine transaminase]) were performed. A 12-lead ECG was performed at screening, intensive PK and at the end of the study. Safety bloods were repeated at every intensive PK visit and prior to discharge from the study. Laboratory and clinical abnormalities were graded for severity according to the U.S National Institutes for Health Division of AIDS table for grading severity of adult and pediatric adverse events.

### Pharmacokinetic analysis.

Dolutegravir blood samples were collected in ethylenediaminetetraacetic acid (K_2_EDTA)-coated tubes (17). Samples for artemether/dihydroartemisinin, lumefantrine/desbutyl-lumefantrine, and amodiaquine/desethylamodiaquine were collected in lithium heparin tubes, whereas artesunate/dihydroartemisinin was collected in fluoride-oxalate tubes to minimize *ex vivo* degradation of artemisinins to dihydroartemisinin by plasma esterases (18, 19). Blood samples were delivered within 15 min of collection to the laboratory for separation and storage at −80°C until shipment to the Liverpool Bioanalytical Facility and Mahidol University for quantification of dolutegravir and ACTs, respectively. Both laboratories participate in external quality assurance programs for antiretrovirals (Association for Quality Assessment in Therapeutic Drug Monitoring and Clinical Toxicology [KKGT], The Netherlands) and antimalarials (Quality Assurance/Quality Control proficiency testing program supported by the Worldwide Antimalarial Resistance Network) and operate to Good Clinical Practice with assays validated according to published FDA guidelines.

Dolutegravir was extracted using liquid-liquid extraction and analyzed using validated reversed phase liquid chromatography-tandem mass spectrometry (LC-MS/MS) with a lower limit of quantification (LLOQ) set at 10 ng/ml and precision of 5% at low quality control (30 ng/ml) (17).

Antimalarial drugs were extracted using solid-phase extraction and quantified by LC-MS/MS. For artemether and dihydroartemisinin the total-assay coefficients of variation were <6% with an LLOQ of 1.14 ng/ml. For artesunate and dihydroartemisinin, the total-assay coefficients of variation were <7% with LLOQs of 0.119 ng/ml (AS) and 0.196 ng/ml (DHA) (19). For lumefantrine and desbutyl-lumefantrine, the total-assay coefficients of variation were <6% with LLOQs of 7.77 ng/ml (LF) and 0.81 ng/ml (DBL) (9). For amodiaquine and *N*-desethylamodiaquine, the total-assay coefficients of variation were <8% with LLOQs of 0.86 ng/ml (AQ) and 1.13 ng/ml (DEAQ).

### Statistical analysis.

Pharmacokinetic parameters, including the area under the concentration-time curve to the last measurable time point (AUC_0–_*_t_*), terminal elimination half-life (*t*_1/2_), maximum concentration (*C*_max_), and time to *C*_max_ (*T*_max_), were estimated using noncompartmental analysis (WinNonlin, Phoenix, version 6.1; Pharsight, Mountain View, CA). The elimination clearance (CL/F) of the parent drug was calculated using the equation dose/AUC_0–_*_t_*. PK data were log transformed to calculate the GMR, with 90% CI values evaluated using paired (study A) or unpaired (study B) *t* tests and backtransformed to absolute ng/ml concentrations. The changes in PK parameters were considered statistically significant for a drug-drug interaction when the CI did not cross the value of one. An ANOVA was performed by SPSS (Windows standard version 22; SPSS, Inc., Chicago, IL) on PK parameters (AUC_0–_*_t_*, *C*_max_, and *C*_24_) using generalized linear models procedures to assess potential sequence and period-related effects.

## Supplementary Material

Supplemental file 1
