# Supplementary material for: Drug Interactions between Dolutegravir and Artemether-Lumefantrine or Artesunate-Amodiaquine
Source: Antimicrob Agents Chemother. 2019 Jan 29;63(2):e01310-18. doi: 10.1128/AAC.01310-18 (PMC6355558; doi:10.1128/AAC.01310-18)
Supplement: Supplemental file 1 [file 0b57b44c8768f3a81df279641887f4ef_AAC.01310-18-s0001.pdf]

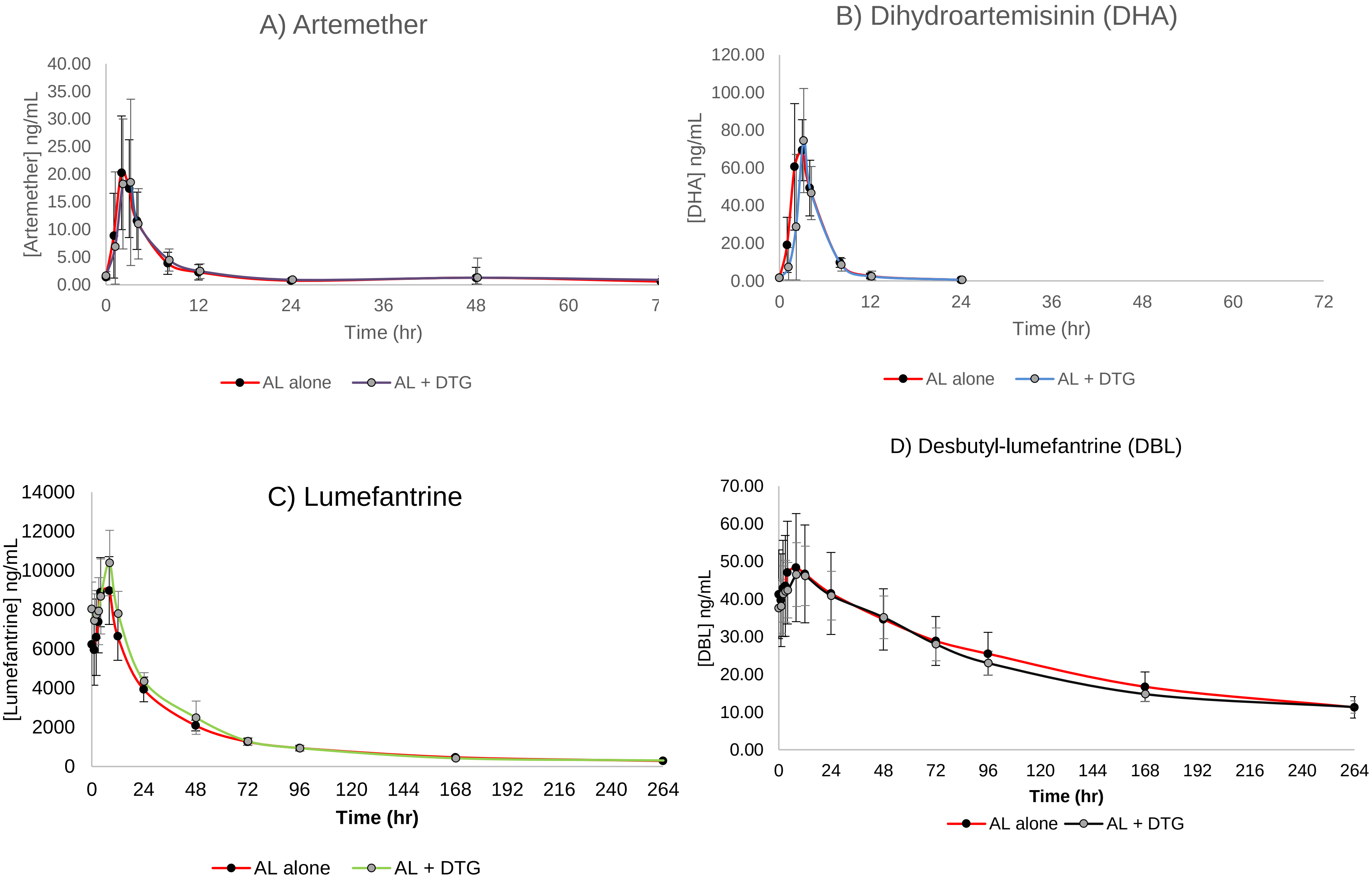

FIG S1. Artemether-lumefantrine parent and active metabolite PK

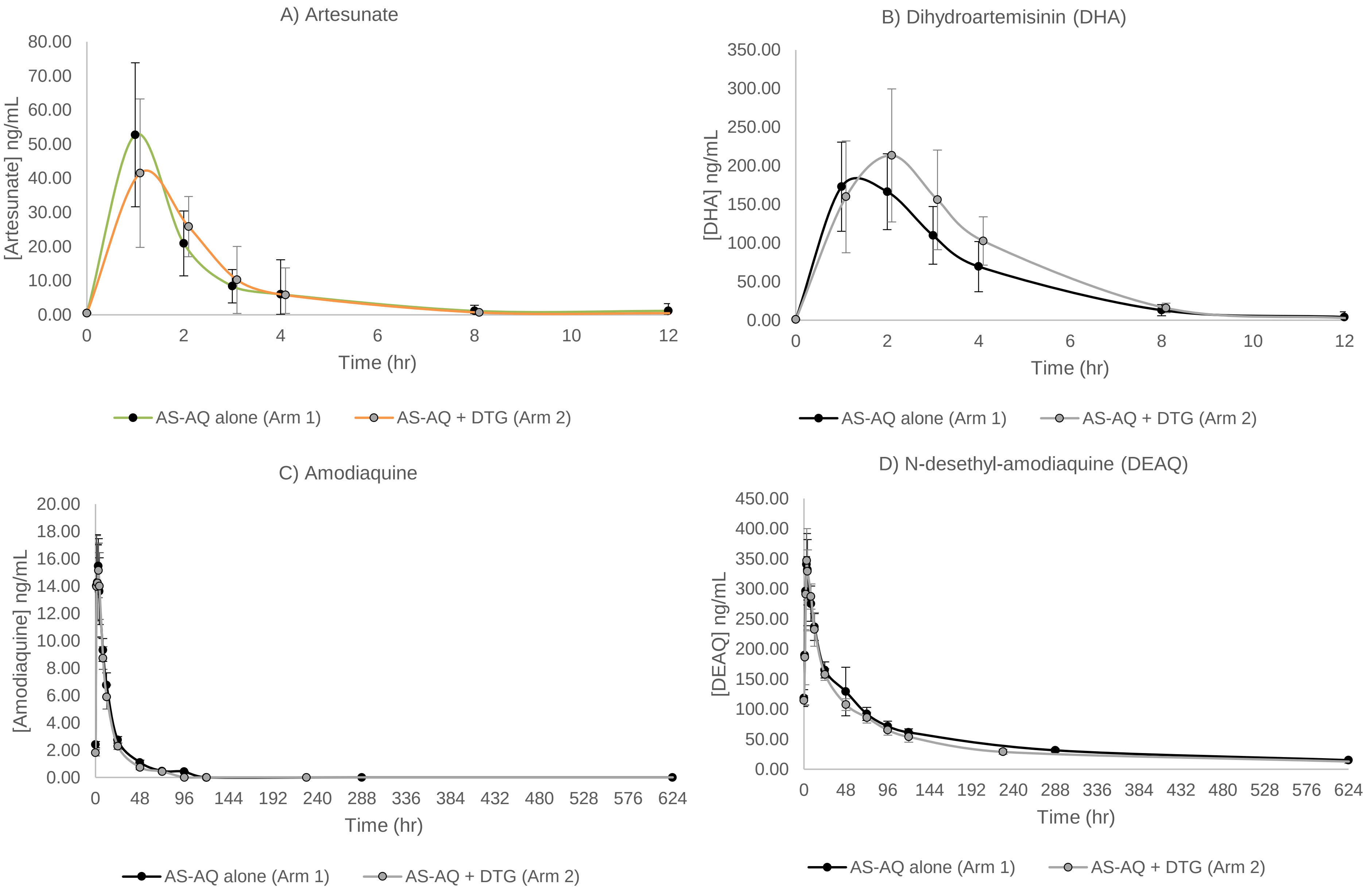

FIG S2. Artesunate-amodiaquine parent and active metabolite PK
